# Impact of genomic and epigenomic alterations of multigene on a multicancer pedigree

**DOI:** 10.1002/cam4.7394

**Published:** 2024-07-05

**Authors:** Jinyu Gao, Yongzhang Wu, Jieming Yu, Yinbin Qiu, Tiantian Yi, Chaochao Luo, Junxiao Zhang, Gary Lu, Xu Li, Fu Xiong, Xuedong Wu, Xinghua Pan

**Affiliations:** ^1^ Department of Pediatrics Nanfang Hospital, Southern Medical University Guangzhou China; ^2^ Guangdong Provincial Key Laboratory of Single Cell Technology and Application Southern Medical University Guangzhou China; ^3^ Department of Biochemistry and Molecular Biology School of Basic Medical Sciences, Southern Medical University Guangzhou China; ^4^ Affiliated Shenzhen Maternity and Child Healthcare Hospital, Southern Medical University Shenzhen China; ^5^ SequMed Institute of Biomedical Sciences Guangzhou China; ^6^ Department of Fetal Medicine and Prenatal Diagnosis Zhujiang Hospital, Southern Medical University Guangzhou China; ^7^ Kaiser Permanente Regional Genetics Laboratory, San Jose Medical Center San Jose California USA; ^8^ Department of Medical Genetics School of Basic Medical Sciences, Southern Medical University Guangzhou China; ^9^ Precision Regenerative Medicine Research Centre, Division of Medical Sciences Macau University of Science and Technology Macao China

**Keywords:** CFH, DNA methylation, DNAH11, familial cancer syndromes, germline mutations, somatic mutation, whole‐exome sequencing, whole‐genome sequencing, WT1

## Abstract

**Background:**

Germline mutations have been identified in a small number of hereditary cancers, but the genetic predisposition for many familial cancers remains to be elucidated.

**Methods:**

This study identified a Chinese pedigree that presented different cancers (breast cancer, BRCA; adenocarcinoma of the esophagogastric junction, AEG; and B‐cell acute lymphoblastic leukemia, B‐ALL) in each of the three generations. Whole‐genome sequencing and whole‐exome sequencing were performed on peripheral blood or bone marrow and cancer biopsy samples. Whole‐genome bisulfite sequencing was conducted on the monozygotic twin brothers, one of whom developed B‐ALL.

**Results:**

According to the ACMG guidelines, bioinformatic analysis of the genome sequencing revealed 20 germline mutations, particularly mutations in the DNAH11 (c.9463G > A) and CFH (c.2314G > A) genes that were documented in the COSMIC database and validated by Sanger sequencing. Forty‐one common somatic mutated genes were identified in the cancer samples, displaying the same type of single nucleotide substitution Signature 5. Meanwhile, hypomethylation of PLEK2, MRAS, and RXRA as well as hypermethylation of CpG island associated with WT1 was shown in the twin with B‐ALL.

**Conclusions:**

These findings reveal genomic alterations in a pedigree with multiple cancers. Mutations found in the DNAH11, CFH genes, and other genes predispose to malignancies in this family. Dysregulated methylation of WT1, PLEK2, MRAS, and RXRA in the twin with B‐ALL increases cancer susceptibility. The similarity of the somatic genetic changes among the three cancers indicates a hereditary impact on the pedigree. These familial cancers with germline and somatic mutations, as well as epigenomic alterations, represent a common molecular basis for many multiple cancer pedigrees.

## 1 INTRODUCTION

Cancer is typically viewed as a somatic disease that arises sporadically, yet numerous cases of familial cancers have been documented. Individuals with a family history of cancer face a cancer risk at least twofold higher than those without affected relatives.^1^ Both germline and somatic genetic variations play distinct roles in cancer initiation and progression.^2^ Specific germline genomic alterations can heighten an individual's susceptibility to cancer.^3^, ^4^, ^5^ For instance, a germline mutation in P53 can lead to Li‐Fraumeni syndrome, characterized by a predisposition to multiple cancers. Pathogenic genomic variants in BRCA1 and BRCA2 significantly elevate the lifetime risk of breast and ovarian cancer.^6^, ^7^ Up to now, over 200 hereditary cancer syndromes have been identified,^8^ and each associated with a range of germline alterations. While a series of rare germline cancer‐susceptible single nucleotide variations (SNVs) and copy number alternations (CNVs) have been pinpointed through extensive cancer sequencing,^3^, ^9^ deciphering and interpreting most multicancer pedigrees remains a challenge, where the carcinogenesis may be impacted by multigenes with minor effects.

In the realm of somatic mutations, a sequential cascade of alterations often occurs in specific well‐defined genes, disrupting crucial signaling pathways.^5^ A distinct pattern of somatic genomic aberrations, notably SNVs, serves as a hallmark of many cancers.^10^ Cancer development is linked to the accumulation of mutations in the genome, giving rise to characteristic mutational signatures.^11^ These signatures offer insights into both internal and external oncogenic factors tied to DNA damage and repair processes.^12^

Beyond genetic predisposition, research among twins suggests that the non‐genetic component, estimated at 60%–70%, plays a significant role through changes in the epigenome, including DNA methylation, influenced by external factors.^13^, ^14^ DNA methylation governs gene activity through various mechanisms, with reports indicating its involvement in the dysregulation of cell cycle control via the methylation of tumor suppressor gene promoters, ultimately driving cancer progression.^15^ Hypermethylation of cancer suppressor genes such as RB1, CDKN2A, MLH1, VHL, and BRCA1, leading to their repression, has been observed in various cancer types.^16^

In this study, a pedigree spanning three generations with three different cancers was examined to identify germline predisposition patterns and a profile of somatic mutations. Additionally, DNA methylation alterations were detected in a panel of cancer‐associated genes in a pair of monozygotic twins, one of whom developed B‐ALL. These findings shed light on common molecular profiles associated with familial predisposition and aim to enhance our understanding of the molecular mechanisms underlying cancer, paving the way for further research in this field.

## 2 MATERIALS AND METHODS

### 2.1 Patients and specimens

The patients hailed from a family in Lufeng City, Guangdong Province, China. The research project received approval from the Ethics Committee of Southern Hospital. Prior to participation, all individuals or relevant guardians of the children within the family provided informed consent and duly signed the consent form. Specimens, including peripheral blood, biopsies from patients with breast cancer (BRCA) or adenocarcinoma of the esophagogastric junction (AEG), and bone marrow from patients with acute lymphoblastic leukemia (B‐ALL) at diagnosis and during remission, were collected.

### 2.2 Hematoxylin and eosin (H&E) staining of BRCA and AEG tissues

The tissue specimens were prepared by fixing each sample in 10% formaldehyde at 4°C for 24 h before embedding them in paraffin. Subsequently, the FFPE tissue blocks were sectioned, placed on glass slides, and air‐dried. For experimental analysis, each section underwent dewaxing, rehydration through an ethanol series, and H&E staining at room temperature. The stained slides were then scanned using an Olympus light microscope at 40×, 100×, or 200×, with evaluation conducted by two pathologists.

### 2.3 DNA extraction, WES, WGS, WGBS library preparation, and sequencing

DNA was extracted from peripheral blood and biopsy tissues utilizing a TIANamp Genomic DNA Kit (cat. no. 4992254, TIANGEN Biotech, Beijing, China) following the manufacturer's instructions. A high‐performance ultrasonic sample processing system (Covaris, Woburn, MA, USA) was employed to randomly fragment the DNA, with fragments ranging from 300 to 350 bp in length recovered for library construction. The DNA fragments underwent end repair, A‐tailing, and adapter ligation for WGS library preparation. Whole‐genome sequencing (WGS) was performed on the BGISEQ‐500 platform. The whole exome was captured using the xGen® Exome Research Panel v1.0 (Integrated DNA Technologies, Coralville, IA, USA) and sequenced on the Illumina NovaSeq 6000 (Illumina, San Diego, CA, USA). For whole‐genome bisulfite sequencing (WGBS) library preparation, an EZ DNA Methylation‐Gold™ Kit (cat. no. D5006; Zymo Research, Irvine, CA, USA) was utilized, followed by sequencing on an Illumina HiSeq 3000 platform.

### 2.4 Bioinformatics analysis

#### 2.4.1 Quality control

The WGS and WGBS data involved utilizing a common Fastq sequencing data quality control method. The raw data in FASTQ format underwent initial processing through the processing tool fastp (v0.23.0). Subsequently, metrics such as Q20, Q30, and GC contents were calculated to ensure the generation of clean, high‐quality data for downstream analyses.

#### 2.4.2 SNVs mapping, variant calling, annotation, and scoring

The WGS and WES data were aligned to the human reference genome University of California Santa Cruz (UCSC) hg19 using the Burrows–Wheeler Aligner (BWA‐MEM2). SAMtools (https://github.com/samtools/samtools) was employed to convert the resulting SAM files to BAM files. Genome Analysis Toolkit (GATK) best practices were utilized for both germline and somatic SNV calling, with HaplotypeCaller used for germline SNVs and Mutect2 for somatic SNVs, generating a Variant Call Format (VCF) file. Additionally, a local normal control library was constructed using WES data from 50 patients with Williams syndrome and WGS data from families NA12878, NA12891, and NA12892 from the 1000 Genomes Project.

For SNVs identified from matched WGS and WES data of the same individuals, the BCFtools merge function was applied for integration. Subsequent processing involved using Bedtools norm to split multiallelic variants into individual variants per line. The variants were annotated using ANNOVAR and Ensembl Variant Effect Predictor (VEP), with genetic variant classification performed using InterVar and Varsome. Population frequency values were determined by selecting the highest value between the genomAD genome EAS and the genomAD exome EAS. SNVs were annotated with the avsnp150 database, and disease‐related gene databases such as ClinVar, OMIM, HPO, and ClinGen were consulted for references.

Comprehensive assessment results from pathogenicity prediction software tools, including REVEL and Varcards, were integrated. Thirteen and 23 in silico algorithms or tools, respectively, were used to predict the pathogenicity of alternative splicing. A pathogenicity‐weighted scoring system based on the ACMG classification guidelines, pathogenicity prediction software, patterns of inheritance, population frequency, mutation location importance, and SNV quality was applied to evaluate the significance of each SNV. Further details on the scoring system can be found in Table S1.

#### 2.4.3 Filtering familial germline variants

Germline mutations shared by MTF_22, MTF_23, MTF_35, and MTF_43 were screened, while MTF_36 and MTF_44 were not restricted. The screening results were further refined based on the cancer‐related gene databases (COSMIC, IntOGen, NCG, and OncKB) and ranked by score from the weighted scoring system (Table S1).

#### 2.4.4 Filtering somatic variants of three cancer samples

The following mutations were identified in the cancer tissue but not in the paired tissue samples of three cancer patients: MTF_22T/MTF_22, MTF_23T/MTF_23, and MTF_43T/MTF_43. The screening results were further refined based on the cancer‐related gene databases (COSMIC, IntOGen, NCG, and OncoKB) and ranked by score from the weighted scoring system.

### 2.5 Mapping, annotation, and filtering for germline CNVs and somatic CNVs

CNVpytor was utilized to identify CNVs, and ClassifyCNV was employed for CNV annotation based on WGS sequencing data. CNVs were excluded if classified as benign or likely benign by ClassifyCNV or if the “CNV level” exceeded three. CNVs were considered common somatic CNVs when shared among all three patients, MTF_22T, MTF_23T, and MTF_43T, with over 50% of the region overlapping, and when the CNV contained at least one protein‐encoding gene.

### 2.6 Mapping, annotation, and screening of DNA methylation data

Sequencing data were mapped to the UCSC human reference genome (hg19) using Bismark (v 0.22.1). The R package DSS (v2.50.0) was utilized for the annotation of differentially methylated CpGs (DMCs), differentially methylated regions (DMRs), and differentially methylated genes (DMGs). The hypermethylated genes were compared to cancer databases (COSMIC, IntOGen, and NCG).

### 2.7 Gene functional analysis

Gene Ontology (GO) pathway enrichment was investigated using the “clusterProfiler” package (v4.10.0). The SNV signature was analyzed by the R package “MutationalPatterns” (v3.12.0) for mutational signature analysis. Tissue‐specific gene expression was determined via GTEx (https://www.gtexportal.org). The following cancer‐associated gene databases were utilized: COSMIC (https://cancer.sanger.ac.uk/cosmic, v98), IntOGen (https://www.intogen.org/search, version: 2023.05.31), and NCG (http://www.network‐cancer‐genes.org, Release 7.1). OncoKB (https://www.oncokb.org/cancerGenes, release date 2023.07.28) was employed for SNV screening. GeneMANIA (http://genemania.org) was used for visualizing protein relationships within a set of input gene sets and interaction networks. Cohort data of methylation, mRNA expression, and survival of B‐ALL were obtained from database TARGET (https://www.cancer.gov/ccg/research/genome‐sequencing/target) and cBioPortal.^17^

## 3 RESULTS

### 3.1 Description of the pedigree and overall sequencing data

**FIGURE 1:**
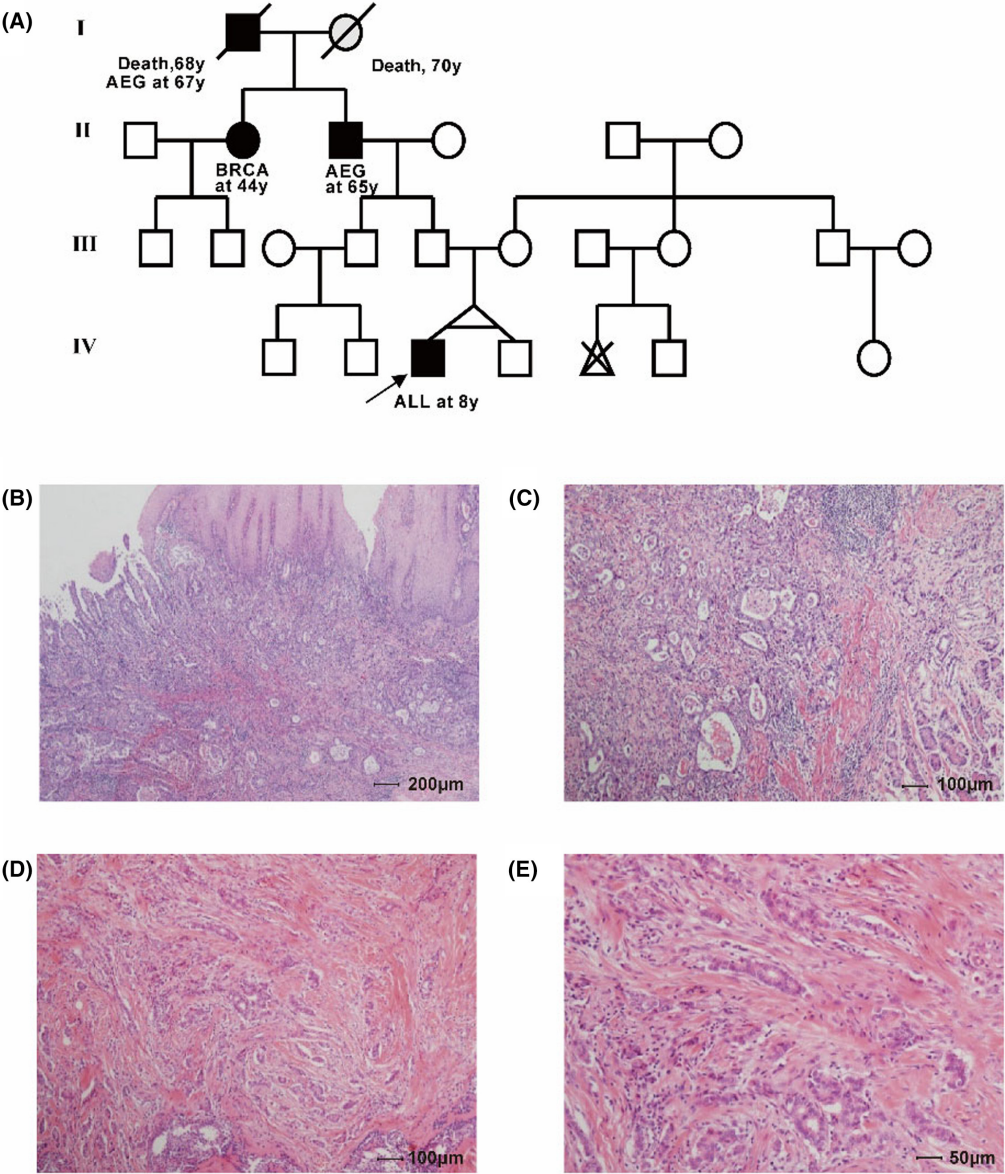
Pedigree chart and H&E staining of the tumor biopsies. (A) Pedigree chart: Males are represented by squares and females by circles. Black symbols denote patients diagnosed with cancer, white symbols represent individuals without cancer, and gray symbols indicated an unknown cancer condition. Symbols with diagonal lines represented deceased individuals. Patient II‐2: BRCA (breast cancer); Patient II‐3: AEG (adenocarcinoma of the esophagogastric junction); Patient IV‐3: B‐ALL (acute lymphoblastic leukemia). (B) and (C) H&E‐stained images of AEG from Patient II‐3 at resolutions of 40× and 100×. (D) and (E) Images of H&E‐stained breast cancer tissue from Patient II‐2 at resolutions of 100× and 200×.

**FIGURE 2:**
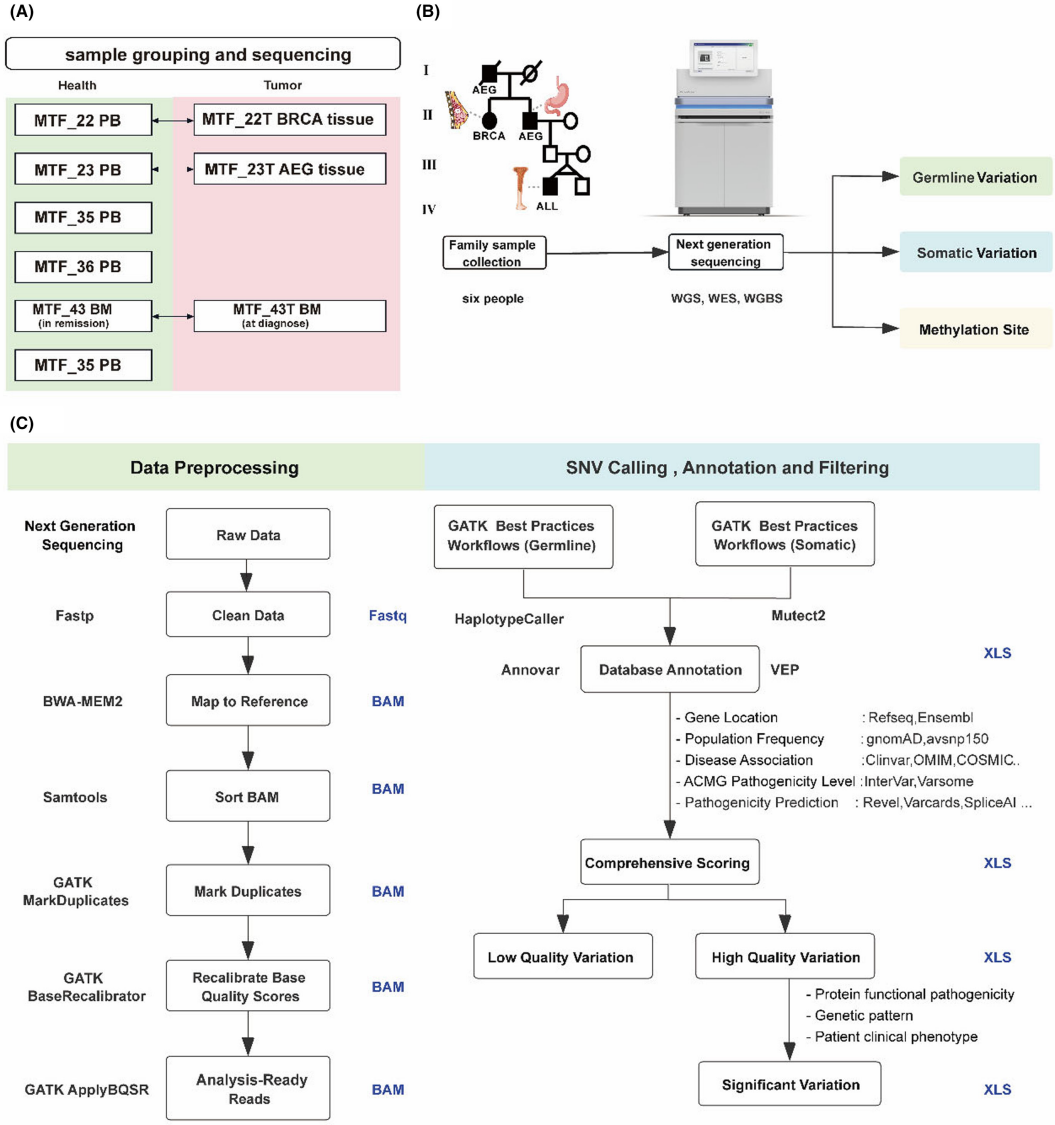
Samples with sequencing information and bioinformatic analysis workflow diagram (A) DNA samples from six healthy family members and three tumor specimens (II‐2, II‐3, III‐5, III‐6, IV‐3, and IV‐4). PB, peripheral blood mononuclear cell (PBMC); BM, bone marrow mononuclear cell (BMMC); AEG, adenocarcinoma of the esophagogastric junction. (B) Experimental and sequencing workflow. (C) Bioinformatic workflow for data preprocessing, SNV calling, annotation and filtering. BAM, binary alignment map format; SNV, single nucleotide variant; XLS, table format.

In this multicancer pedigree, three affected individuals (II‐2, II‐3, and IV‐3) and three unaffected individuals (III‐5, III‐6, and IV‐4) were identified (Figure 1A). Proband IV‐3, a child diagnosed with B‐acute lymphoblastic leukemia (B‐ALL) at 8 years old, tested positive for ETV6::RUNX1 fusion (Figure S1). IV‐4, the healthy monozygotic twin brother of IV‐3, exhibited homozygosity as revealed by STR analysis of WGS data using SAMtools and IGV based on the NIST Standard Reference Database with 20 core STR loci of the Combined DNA Index System (CODIS). III‐5 and III‐6, the father and mother of IV‐3, respectively, were in good health with no identified tumors. II‐3, the grandfather of IV‐3, had AEG at pT2N2M0 diagnosed at Age 65 and was currently in remission post‐surgical resection. II‐2, the sibling of II‐3, a female diagnosed with stage IIA (pT1N1M0) breast cancer at 44 years old according to the American Joint Committee on Cancer (AJCC), was also in remission. Pathologic sections II‐2 and II‐3 were confirmed by pathologists (Figure 1B–E). Specimens collected included peripheral blood and BRCA biopsy from II‐2, peripheral blood and AEG biopsy from II‐3, bone marrow samples of B‐ALL at diagnosis and in remission from IV‐3, and peripheral blood samples from healthy family members III‐5, III‐6, and IV‐4 (Figure 1A and Figure 2A). Genomic analysis was conducted on three affected individuals (II‐2, II‐3, and IV‐3) and three unaffected individuals (III‐5, III‐6, and IV‐4) to study the molecular pathogenesis of the cancer family (Figure 2A). WGS was performed on eight samples (Table 1, Table S2). In noncancer specimens, the mean depth was 42.62× (range 41.43 × −46.05×), with an average coverage of over 10× at 91.84% (range 91.42%–92.09%). In cancer specimens, the mean depth was 78.51× (range 67.18 × −89.04×), with an average coverage of over 20× at 91.44% (range 90.24%–92.06%) (Figure 2B).

**TABLE 1:** Pedigree and sequencing information.

Family member	Age, years	Sex	Sample ID	Sample type	Sequencing methods
II‐2	44	Female	MTF_22	Peripheral blood	WGS
			MTF_22T	Breast cancers biopsies	WGS
II‐3	65	Male	MTF_23	Peripheral blood	WGS
			MTF_23T	AEG biopsies	WGS
III‐5	37	Male	MTF_35	Peripheral blood	WGS, WES
III‐6	36	Female	MTF_36	Peripheral blood	WGS, WES
IV‐3	8	Male	MTF_43	Bone marrow at remission	WGS, WGBS
			MTF_43T	Bone marrow at diagnosis	WGS, WES
IV‐4	8	Male	MTF_44	Peripheral blood	WGS, WES, WGBS

### 3.2 Germline SNVs and CNVs

**FIGURE 3:**
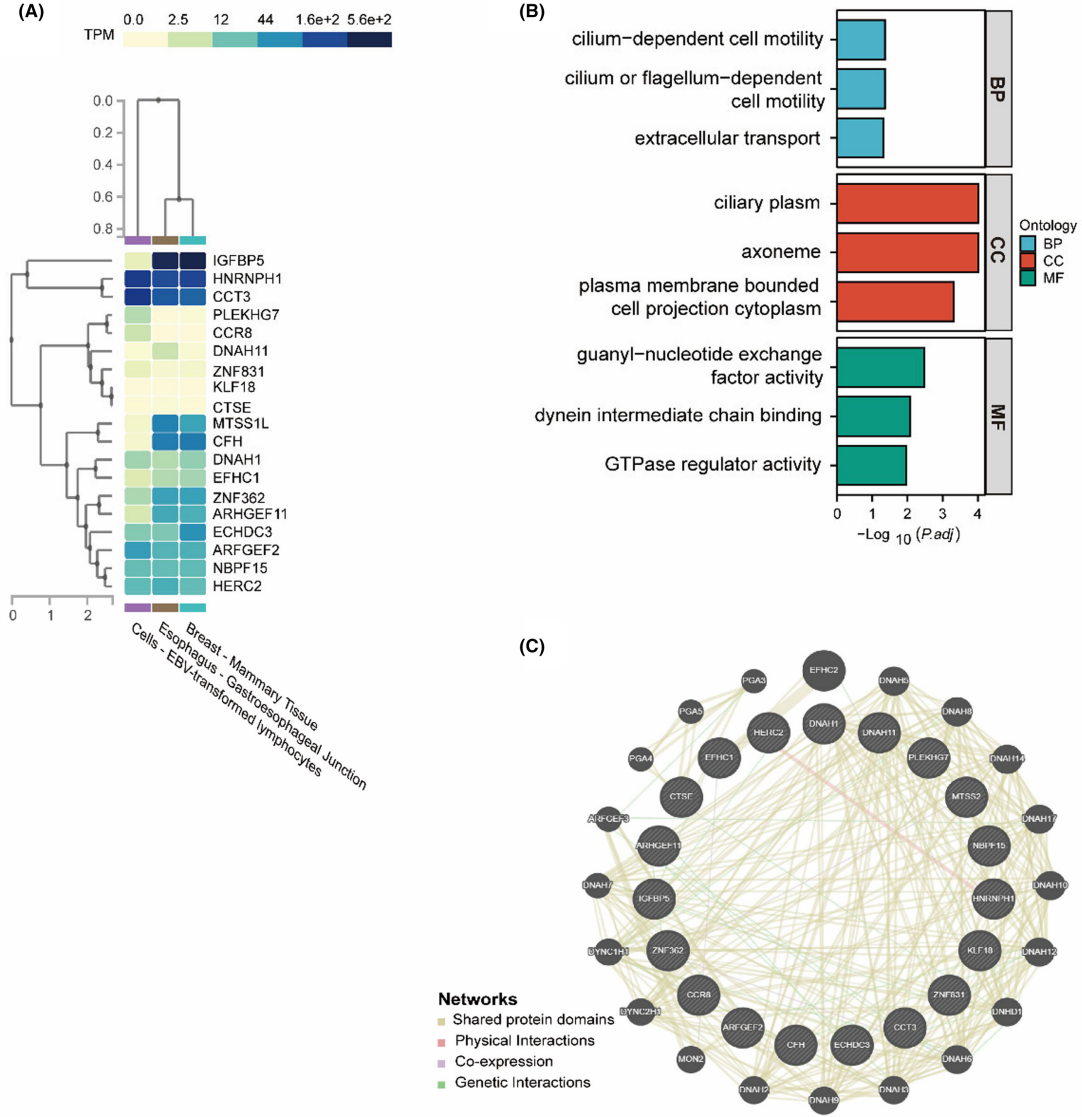
Germline‐mutated genes of the pedigree. (A) Tissue‐specific gene expression of 19 mutated genes in the Genotype‐Tissue Expression Database (GTEx) of healthy tissue. (B) The functional pathway was enriched in cilium‐dependent cell motility (DNAH11/EFHC1/DNAH1) and guanyl‐nucleotide exchange factor activity (ARFGEF2/PLEKHG7/HERC2/ARHGEF11). (C) The GeneMANIA protein–protein interaction network predicts a physical interaction between HERC2 and HNRNPH1. (B) and (C) Germline SNVs were derived from three members of the pedigree (MTF_23, MTF_35, and MTF_43), while 20 sites in 19 genes with high scores were cancer related.

To identify germline single nucleotide variants (SNVs), we conducted mutation screening and identification following the methods outlined in the methods section (Figure 2C). A pathogenicity weight scoring system for germline and somatic SNVs was established based on the ACMG classification (Table S1). After implementing the filtering procedure, we identified 261 associated SNVs shared among three members of the pedigree—the grandfather, father, and proband (MTF_23, MTF_35, and MTF_43). Through weighted scoring, we pinpointed 20 SNVs in 19 genes with higher scores that were deemed cancer‐related (Table 2, Tables S3 and S4). However, all variants were classified as variants of uncertain significance according to the ACMG guidelines. Analysis of the GTEx database revealed that over 60% of these genes exhibited expression in normal cells of the three cancer types, indicating their functional significance in normal tissue (Figure 3A). Gene function analysis indicated enrichment in cilium‐dependent cell motility (DNAH11/EFHC1/DNAH1) and guanyl‐nucleotide exchange factor activity (ARFGEF2/PLEKHG7/HERC2/ARHGEF11) (Figure 3B), suggesting potential synergistic effects of mutations in these genes. Notably, apart from the confirmed physical interaction between HERC2 and HNRNPH1, no direct protein interactions were observed among the other genes (Figure 3C).

**TABLE 2:** Germline mutation loci identified in the pedigree.

Gene symbol	HGVS	dbSNP ID	Mutation type	Exon	VarCards	COSMIC ID
DNAH11	NM_001277115.2: c.9463G > A: p.Ala3155Thr	rs752191917	Missense variant	57/82	20:23	COSV60941441
CFH	NM_000186.4: c.2314G > A: p.Asp772Asn	rs374704701	Missense variant	15/22	1:22	COSV66408069

Abbreviations: CFH, complement factor H; COSMIC, cancer SNV database; dbSNP, single nucleotide polymorphism database (dbSNP); DNAH11, Dynein Axonemal Heavy Chain 11; HGVS, Human Genome Variation Society; VarCards: tools of protein damage prediction tools (number of tools that predicted SNV as protein function damage: number of prediction tools used).

To further identify cancer‐predisposing SNVs, we screened COSMIC‐documented loci, leading to the identification of the SNVs DNAH11 c.9463G > A (p.Ala3155Thr) and CFH c.2314G > A (p.Asp772Asn) in the COSMIC database. Sanger sequencing validated these mutations in six pedigree members (MTF_22, MTF_23, MTF_35, MTF_36, MTF_43, and MTF_44) (Table 2, Figure S2). VarCards prediction tool analysis revealed that 20 out of 23 tools predicted DNAH11 c.9463G > A to be deleterious to protein function. According to COSMIC, the DNAH11 variant is associated with esophageal carcinoma, while CFH is linked to breast cancer. DNAH11, a member of the dynein heavy chain family encoding a ciliary outer dynein arm protein, has been implicated as a predisposition gene in breast cancer families.^18^, ^19^ Additionally, mutations in DNAH11 have been reported in BRCA1‐ and BRCA2‐negative breast cancer families,^20^ further supporting its role in cancer predisposition. DNHA11 is one of 42 genes that shared conserved response to hypoxia,^21^ which indicates that DNHA11 mutation might deregulate cell response of hypoxia. CFH is required to maintain cancer stemness via late SV40 factor that regulates thymidylate synthase gene, thus contributing to cell cycle regulation.^22^, ^23^ Furthermore, four mutated genes in the family are found in the NCG database: HERC2, ZNF831, ARHGEF11, and ZNF362. HERC2, belonging to the HERC family of ubiquitin ligases, plays a crucial role in cellular processes such as the DNA damage response, targeting BARD1‐uncoupled BRCA1 for degradation and potentially compromising DNA repair mechanisms.^24^, ^25^ Therefore, HERC2 mutation potentially triggers dysregulation of DNA double strand break repair.^26^ Study has revealed that ZNF831 knockdown enhances proliferation of breast cancer cells,^27^ suggesting that ZNF831 mutations may contribute to cancer progression. Lastly, to investigate the potential involvement of common copy number variants (CNVs) in the pedigree's carcinogenesis, we performed CNV analysis and annotation for all family members. However, no shared pathogenic germline CNVs exceeding 1 Mb in length were identified across the six samples.

### 3.3 Somatic SNVs and CNVs

**FIGURE 4:**
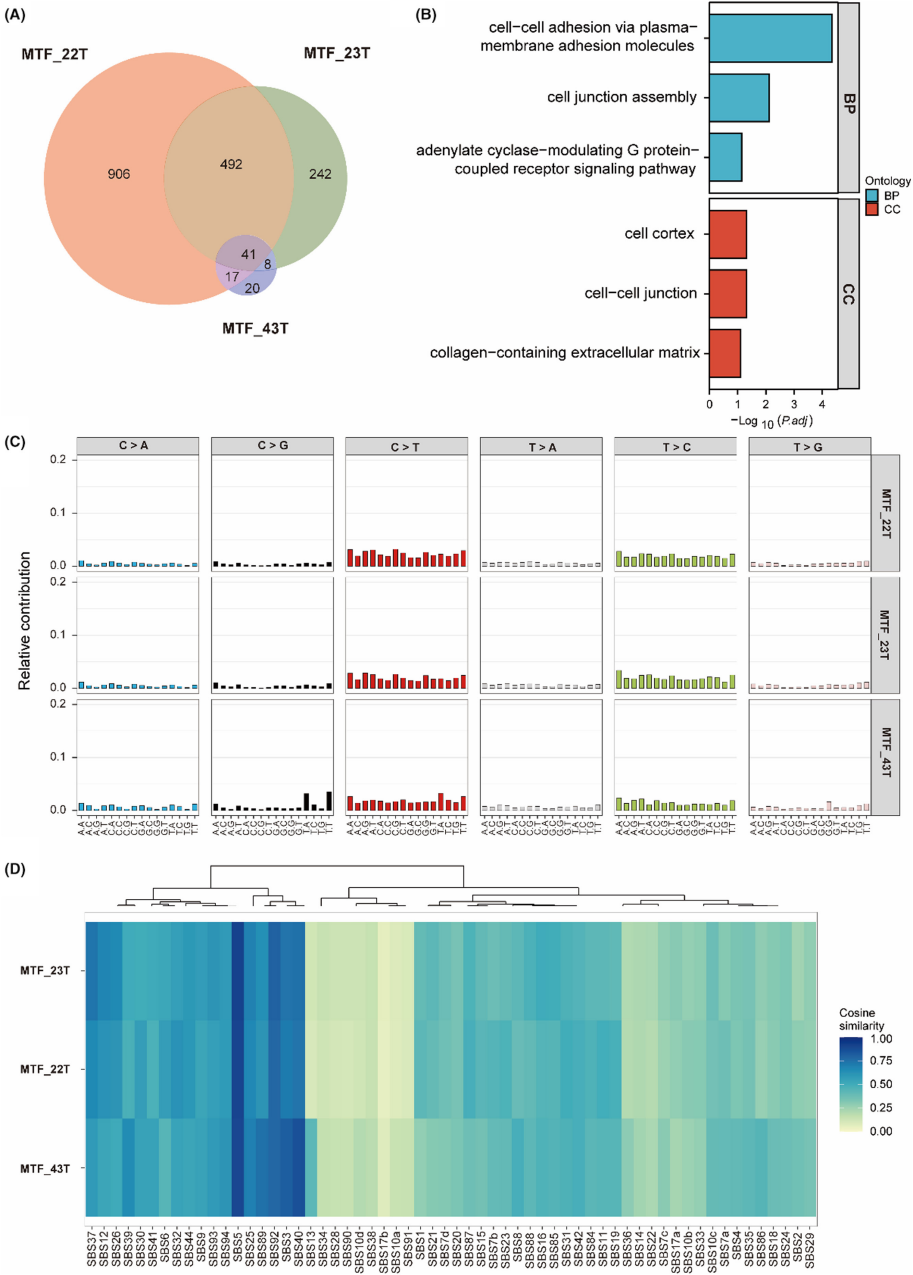
Somatic mutation analysis of SNVs in three tumor samples (MTF_22T, MTF_23T, and MTF_43T). (A) Intersection between SNVs of MTF_22T, MTF_23T, and MTF_43T. (B) Functional pathway of 41 genes with somatic mutations shared by MTF_22T, MTF_23T, and MTF_43T. (C) Single nucleotide substitution (SBS) signature analysis of MTF_22T, MTF_23T, and MTF_43T. The SBS was enriched in the C > T pattern. (D) SBS signature classes of MTF_22T, MTF_23T, and MTF_43T. These parameters characterized SBS Signature 5.

There were 1456, 783, and 86 SNVs found in MTF_22T, MTF_23T, and MTF_43T, respectively, reported in the COSMIC database. Due to tumor heterogeneity, there were intersections and differences among the three tumor samples. Individually specific SNVs comprised 97%, 95%, and 52% of the SNVs in MTF_22T, MTF_23T, and MTF_43T, respectively. In total, 41 SNVs were common in the three samples. These SNVs have cellular junction functions, such as cell–cell adhesion via plasma‐membrane adhesion molecules and cell junction assembly (Figure 4A,B), but none of these common somatic SNVs match the COSMIC report. Interestingly, both MTF_22T and MTF_23T were derived from the epithelium, as shown by 11 COSMIC‐reported SNVs (Table S5), presenting more common somatic changes with carcinogenic risk compared to MTF_43T, a leukemia. Additionally, MTF_23T and MTF_43T shared 11 common somatic SNVs, 3 of which were identified in the COSMIC database (Table S6). Furthermore, MTF_22T, MTF_23T, and MTF_43T also shared two somatic CNV regions encoding protein‐coding genes (Table S7).

The SNVs of MTF_22T are located in genes encoding proteins involved in cancer‐related signaling pathways such as the regulation of the Wnt signaling pathway, Ras signaling pathway, and PI3K‐Akt signaling pathway, while the SNVs of MTF_23T are also found in genes related to similar signaling pathways such as Ras protein signal transduction and the PI3K‐Akt signaling pathway, and MTF_43T to protein tyrosine phosphatase activity, cadherin binding, and phosphoprotein phosphatase activity (Figure S3). Somatic mutational signature analysis of MTF_22T, MTF_23T, and MTF_43T suggested that the characteristics of the three tumors were similar, all identified as SBS Signature 5 (Figure 4C,D). Taken together, these findings suggest that certain common factors lead to susceptibility to different types of cancers according to the pedigree.

### 3.4 Differences in methylation between monozygotic twins

**FIGURE 5:**
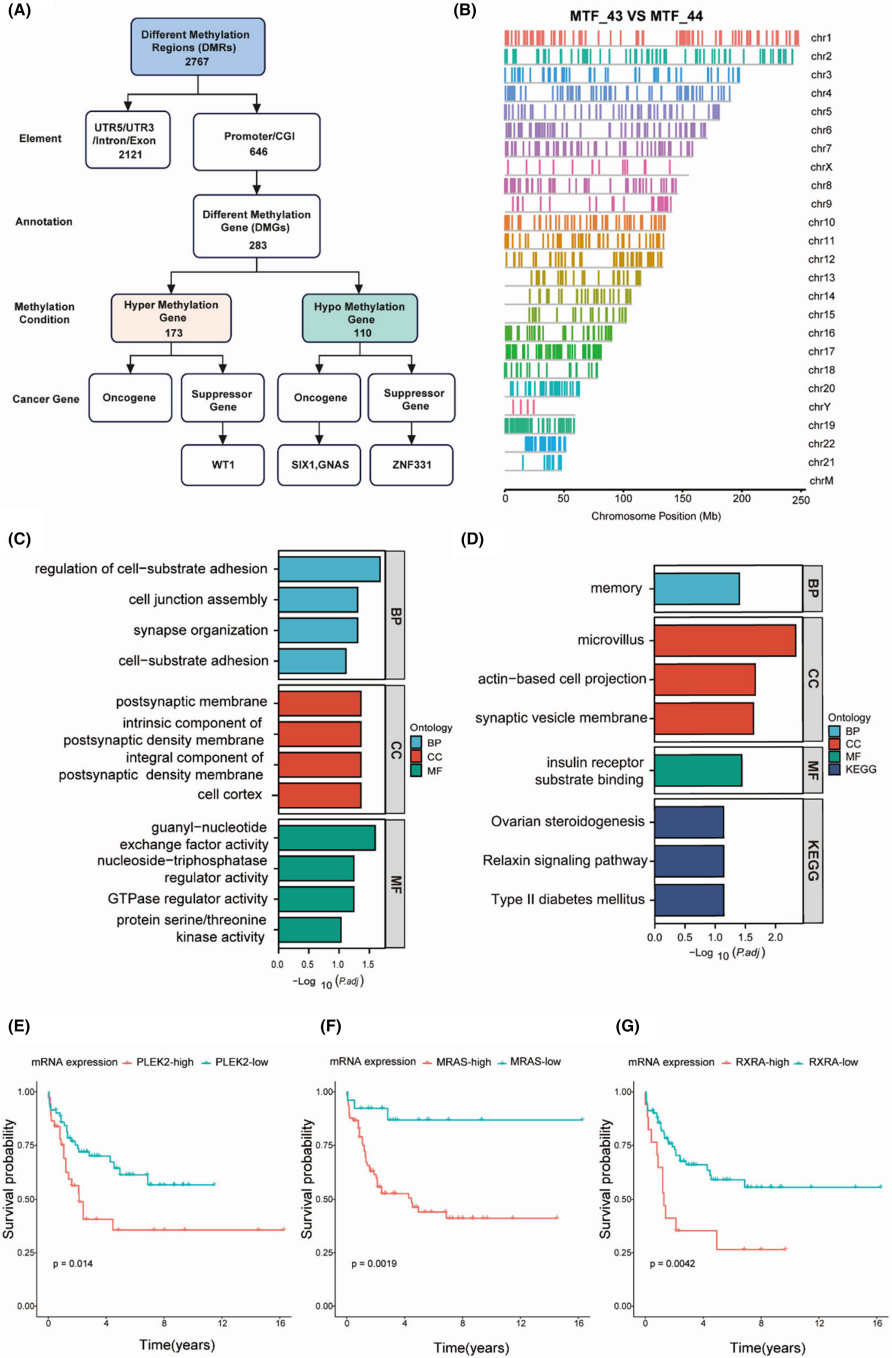
Methylation analysis of the twins MTF_43 and MTF_44. (A) Flowchart for screening for tumor‐associated genes with altered DNA methylation. (B) DMR of MTF_43 vs MTF_44 on all chromosomes. The colors represented different chromosomes. (C) Functional pathway of the hypermethylated genes in MTF_43 compared with MTF_44. (D) Functional pathway of hypomethylated genes in MTF_43 compared with MTF_44. (E–G) The impact of mRNA expression of PLEK2, MRAS, and RXRA on overall survival in pediatric B‐ALL cohort (data from TARGET database, see Section 2).

IV‐3 and IV‐4, who share a common germline mutation background but experience different clinical events, indicate that epigenomic factors, such as methylation, may play an important role. Therefore, whole‐genome bisulfite sequencing (WGBS) was performed on the bone marrow mononuclear cells (BMMC) in remission from the twin with B‐ALL and on the peripheral blood mononuclear cells (PBMC) from the healthy twin (Figure 5A). Despite potential subtle differences between PBMC and BMMC, several investigations have demonstrated and took the advantages of the similarity and comparability of DNA methylation profiles between two tissue sources,^28^ considering the scarcity of BMMC samples from healthy pediatric individuals. Significant differences in methylation were observed across the genome (Figure 5B). The percentages of DNA methylation in the genomic regions of MTF_43 (B‐ALL twin) and MTF_44 (healthy twin) were 81.27% and 81.15%, respectively. Differential methylation was detected in MTF_43 compared to MTF_44: 173 hypermethylated and 110 hypomethylated genes. Cell‐substrate adhesion was enriched among the hypermethylated genes of the B‐ALL twin MTF_43, and the Ras signaling pathway was enriched among the hypomethylated genes of MTF_43 (Figure 5C,D). The CpG island (CGI) of the tumor‐suppressing gene WT1 was hypermethylated in MTF_43 (Table S8). Usually, hypermethylation downregulates gene expression, which might promote B‐ALL in the MTF_43 patient. It has been reported that methylation of WT1 is related to increased breast cancer risk.^29^ Additionally, CGIs or promoters of two oncogenes (SIX1 and GNAS) were also hypomethylated in the MTF_43 cells (Table S8). Promoter methylation of SIX1 was shown to impact its expression, and high expression of SIX1 was reported to activate the Notch signaling pathway and predict poor prognosis in lung cancer patients.^30^, ^31^ Last but not the least, three hypomethylated genes in MTF_43 (B‐ALL twin)—PLEK2, MRAS, and RXRA—display a correlation with mRNA hyperexpression (Figure S4) and mRNA hyperexpression, and they are associated with poor outcomes in pediatric B‐ALL cohort (Figure 5E–G). For instance, PLEK2 has been reported to promote cancer stemness, tumorigenesis, invasion, and metastasis.^32^, ^33^ In addition, MRAS has also been found involving in RAF activation in certain cancers.^34^

## 4 DISCUSSION

One of the consistent goals in cancer research is to understand the genetic variants responsible for cancer development and progression.^35^ The transition from benign lesions to malignant tumors necessitates the gradual accumulation of mutations over time.^2^ Identifying crucial mutations in families with a history of cancer is crucial, as these germline mutations can increase susceptibility to cancer.^36^ Large‐scale studies have been conducted for predisposing variants, revealing pathogenic or likely pathogenic variants in 8% of 33 cancer types in adults and 8.5% of children and adolescents with cancer.^37^, ^38^ Furthermore, the burden of germline variants in cancer genes has been linked to the age at diagnosis and the burden of somatic mutations.^39^

In the present study, three different types of cancer were observed in three different generations of a family, potentially due to specific germline mutations. Using next‐generation sequencing (NGS), 20 germline mutations in 19 cancer‐associated genes were identified, with some genes previously reported in databases like COSMIC and NCG. However, these mutations did not meet the strict criteria for pathogenic variants according to ACMG guidelines. Nonetheless, the functionality of these genes suggests that variations within them may collectively contribute to increased cancer susceptibility through the functional pathway of DNA repair responses, cell cycle regulation, and proliferation.

The lack of cancer development in the father of the proband may be attributed to incomplete penetrance or insufficient cumulative tumor susceptibility, possibly influenced by epigenomic regulation.^40^, ^41^ The epigenetic modifications such as hypermethylation of promoter CpG islands in tumor suppressor genes can impact gene expression patterns and contribute to tumorigenesis.^42^ Meanwhile, hypomethylation of oncogenes may result in transcriptional activation.^43^Hence, epigenetic modification facilitates the development of characteristic tumor traits by controlling gene expression patterns that foster tumorigenesis,^44^ in addition to other mechanisms. Hypermethylation of the tumor suppressor gene WT1 and hypomethylation of two oncogenes (SIX1 and GNAS) were detected in the twin with B‐ALL when in remission. WT1 expression alterations are found in hematological neoplastic disorders including pediatric B‐ALL.^45^, ^46^ The hypomethylation of the genes found in the B‐ALL twin, PLEK2, MRAS, and RXRA, demonstrates a positive association on their mRNA hyperexpression and further impact on adverse outcome in B‐ALL cohort. Epigenetic dysregulation often leads to inappropriate activation or inhibition of signaling pathways, potentially initiating the onset of pathological development, and serving as a predisposing factor for tumorigenesis.^47^ However, further investigations are needed to assess the risk of these methylation alterations in hematological tumorigenesis.^16^

Some SBS signatures have identifiable causes, such as exogenous or endogenous exposures, as well as defective DNA maintenance processes, seen in both adult and pediatric cancer patients. For instance, Signature 3 has a strong correlation with homologous recombination deficiency (HRD).^48^, ^49^ However, many signatures remain of unknown origin.^50^, ^51^ MTF_22T, MTF_23T, and MTF_43T fall under SBS Signature 5, which lacks a specific known cause.^11^ Furthermore, the impact of Signature 5 on mutational signatures in B‐ALL, breast cancer, and gastric cancer patients is minimal in a large‐scale cohort.^11^ The identical signatures among the three cancer patients in this pedigree suggest a hereditary role of SBS Signature 5 in familial cancer.

This study is limited to a single pedigree where multiple family members developed different types of cancers. Nonetheless, our findings indicate that a specific profile of multiple gene mutations may predispose individuals to cancer, though it may not be adequate for definitive tumor initiation. Environmental factors, causing epigenomic modifications, could potentially act as the final trigger for cancer initiation. Further research is needed to elucidate the functional roles of SNVs and dysregulated DNA methylation genes in this context.

In conclusion, a three‐generation family with three members affected by different types of cancer was found to have multiple germline predisposition genes, including DNAH11 and CFH. The tumors exhibited a common set of somatic SNVs and SBS Signature 5. Hypermethylation of TSG WT1 was observed in the monozygotic twin with B‐ALL but not in the healthy twin. These results highlight specific germline and somatic mutations, along with epigenomic influences, in this and potentially other cancer pedigrees. The study proposes a shared molecular scenario for familial cancer, contributing to our understanding of carcinogenesis.

## AUTHOR CONTRIBUTIONS

**Jinyu Gao**: Resources (equal); Formal analysis (equal); writing – original draft (lead). **Yongzhang Wu**: Formal analysis (lead); Data curation (lead); visualization (lead). **Jieming Yu**: Resources (equal). Yinbin Qiu: Software (equal). **Tiantian Yi**: Resources (equal). **Chaochao Luo**: Methodology (equal). **Junxiao Zhang**: Resources (equal). **Gary Lu**: Writing – review and editing (equal). **Xu Li**: Writing – review and editing (equal). **Fu Xiong**: Writing – review and editing (equal). **Xuedong Wu**: Conceptualization (equal); project administration (equal); supervision (equal). **Xinghua Pan**: Conceptualization (equal); Funding acquisition (lead); project administration (equal); supervision (equal); writing – review and editing (lead).

## ETHICS STATEMENT

The Ethics Committee of Southern Hospital approved this research project.

## CONSENT

All participants or the relevant guardians of the children provided informed consent and signed the informed consent form.

## Supporting information


**Data S1:** Supporting Information.

## Data Availability

The WGS raw variation sequencing VCF data supporting this study has been deposited in the Genome Sequence Archive at the National Genomics Data Center, China National Center for Bioinformation/Beijing Institute of Genomics, Chinese Academy of Sciences (https://ngdc.cncb.ac.cn/gsa‐human, accession no. PRJCA022384). Partial custom codes and cell annotation files are accessible at GitHub: https://github.com/leadingsci/Multi‐Cancer_Pedigree_Project. All the other data supporting the findings of this study are available from the corresponding authors upon request.
