# Supplementary material for: Impact of genomic and epigenomic alterations of multigene on a multicancer pedigree
Source: Cancer Med. 2024 Jul 5;13(13):e7394. doi: 10.1002/cam4.7394 (PMC11226725; doi:10.1002/cam4.7394)
Supplement: Supplementary file 1 — Data S1: Supporting Information. [file CAM4-13-e7394-s001.docx]

Supplementary Material

# Supplementary Tables

**Suppl Table 1.** **Pathogenicity Weight Scoring System for germline and somatic SNVs**

| **Score** | **Maximum score** | **Test item** | **Scoring Rule** | **Score** |
| --- | --- | --- | --- | --- |
| Pathogenwtic sites reported early | 5 | Clinvar | Pathogenic | +5 |
|  |  |  | Likely pathogenic | +4 |
|  |  |  | Benign | -5 |
|  |  |  | Likely_benign | -4 |
| Pathogenicity determined by ACMG | 4 | InterVar | Pathogenic | +4 |
|  |  |  | Likely pathogenic | +3 |
|  |  |  | Benign | -4 |
|  |  |  | Likely benign | -3 |
|  |  | InterVar_PS_comprehensive* | recorded | +2 |
|  |  | InterVar_BS_comprehensive# | recorded | -2 |
| Mendel inheritance model | 3 | Inheritance | AR:Het;AR:Het,AR:Hom,AD:Het | +3 |
|  |  |  | AD:Hom | -3 |
|  |  |  | 0.5<REVEL≤0.75 | +2 |
|  |  |  | REVEL<0.15 | -3 |
|  |  | VarCardsL(ACMG PP3) | VarCards≥0.75 | +3 |
|  |  |  | 0.5<VarCards≤0.75 | +2 |
|  |  |  | VarCards<0.15 | -3 |
| Pathogenicity prediction by software (22) | 1 | SIFT_pred | Deleterious | +1 |
|  |  |  | Tolerated | -1 |
|  |  | SIFT4G_pred | Deleterious | +1 |
|  |  |  | Tolerated | -1 |
|  |  | Polyphen2_HDIV_pred | Probably Damaging | +1 |
|  |  |  | Benign | -1 |
|  |  | Polyphen2_HVAR_pred | Probably Damaging | +1 |
|  |  |  | Benign | -1 |
|  |  | MutationTaster_pred | Disease causing | +1 |
|  |  |  | Polymorphism | -1 |
|  |  | dbscSNV_ADA_SCORE | ≥0.6 | +1 |
|  |  |  | <0.6 | -1 |
|  |  | LRT_pred | Disease causing | +1 |
|  |  |  | Polymorphism | -1 |
|  |  | FATHMM_pred | Deleterious | +1 |
|  |  |  | Tolerated | -1 |
|  |  | PROVEAN_pred | Disease causing | +1 |
|  |  |  | Polymorphism | -1 |
|  |  | MetaSVM_pred | Disease causing | +1 |
|  |  |  | Polymorphism | -1 |
|  |  | MetaLR_pred | Deleterious | +1 |
|  |  |  | Tolerated | -1 |
|  |  | MetaRNN_pred | Deleterious | +1 |
|  |  |  | Tolerated | -1 |
|  |  | CAP_pred | Deleterious | +1 |
|  |  |  | Tolerated | -1 |
|  |  | PrimateAI_pred | Deleterious | +1 |
|  |  |  | Tolerated | -1 |
|  |  | DEOGEN2_pred | Deleterious | +1 |
|  |  |  | Tolerated | -1 |
|  |  | BayesDel_addAF_pred | Deleterious | +1 |
|  |  |  | Tolerated | -1 |
|  |  | BayesDel_noAF_pred | Deleterious | +1 |
|  |  |  | Tolerated | -1 |
|  |  | ClinPred_pred | Deleterious | +1 |
|  |  |  | Tolerated | -1 |
|  |  | LIST-S2_pred | Deleterious | +1 |
|  |  |  | Tolerated | -1 |
|  |  | fathmm-MKL_coding_pred | Disease causing | +1 |
|  |  |  | Polymorphism | -1 |
|  |  | fathmm-XF_coding_pred | Disease causing | +1 |
|  |  |  | Polymorphism | -1 |
|  |  | SIFT_pred | Deleterious | +1 |
|  |  |  | Tolerated | -1 |
| Population frequency | 1 | genomAD_EAS (ACMG PM2) | (AR,genomAD_EAS<0.0001) OR (AD,genomAD_EAS<0.001) | +2 |
|  |  |  | genomAD_EAS>0.05 | -2 |
|  |  |  | genomAD_EAS>0.01 | -1 |
| Mutation site location | 1 | Interpro_domain | recorded | +1 |
|  |  | VEP_dbSNPID | recorded | +1 |
|  |  | VEP_Transcript | recorded | +1 |
|  |  | VEP_Exon | recorded | +1 |
|  |  | VEP_Intron | recorded | -1 |
| Genetic disease database | 1 | ACMG_SF_Gene | recorded | +1 |
|  |  | Imprint_Gene | recorded | +1 |
|  |  | OMIM_Gene | recorded | +1 |
|  |  | HPO_Gene | recorded | +1 |
|  |  | ClinGen_Gene | recorded | +1 |
|  |  | cosmic96_coding | recorded | +1 |
|  |  | PseudoGene_Gene | recorded | -1 |
|  |  | genomicSuperDups | recorded | -1 |
| Variation quality | 1 | GATK Quality | Quality>30 | +1 |

InterVar_PS_comprehensive*: S1, PS2, PS3, PS4, PM1, PM2, PM3, PM4, PM5, PM6, PP1, PP2, PP3, PP4, and PP5.

InterVar_BS_comprehensive#: includes BA1, BS1, BS2, BS3, BS4, BP1, BP2, BP3, BP4, and BP5.

**Suppl Table 2. Reads, mapping rate, average depth, and coverage of WGS**

| Sample id |  | Clean bases (Mb) | mapping rate | Average depth (X) | 1X coverage | 10X coverage | 20X coverage |
| --- | --- | --- | --- | --- | --- | --- | --- |
| MTF_22 | WGS | 126329.67 | 99.82% | 42.45 | 91.70% | 91.45% | 90.72% |
| MTF_22T | WGS | 221186.77 | 92.64% | 67.18 | 91.86% | 91.54% | 90.24% |
| MTF_23 | WGS | 123404.82 | 99.76% | 41.43 | 92.35% | 92.04% | 89.21% |
| MTF_23T | WGS | 266506.95 | 99.66% | 89.04 | 92.38% | 92.29% | 92.06% |
| MTF_35 | WGS | 125422.67 | 99.75% | 42.10 | 92.35% | 92.06% | 89.43% |
| MTF_36 | WGS | 123977.77 | 99.82% | 41.66 | 91.69% | 91.43% | 90.54% |
| MTF_43 | WGS | 125095.39 | 99.74% | 42.02 | 92.34% | 91.98% | 89.06% |
| MTF_43T | WGS | 236433.80 | 99.73% | 79.32 | 92.36% | 92.23% | 92.03% |
| MTF_44 | WGS | 137189.24 | 99.74% | 46.05 | 92.35% | 92.09% | 90.19% |

**Suppl** **Table 3. COSMIC reported germline mutation loci (SNVs) in 5 members of pedigree (MTF_22, MTF_23, MTF_35, MTF_43, and MTF_44)**

| **Gene_Symbol** | **Chr_Start_Ref_Alt** | **HGVS** | **dbSNPID** | **Mutation_Type** | **Exon** | **VarCards** | **COSMIC** | **MTF_22** | **MTF_23** | **MTF_35** | **MTF_36** | **MTF_43** | **MTF_44** |
| --- | --- | --- | --- | --- | --- | --- | --- | --- | --- | --- | --- | --- | --- |
| DNAH11 | chr7:21818702:G:A | NM_001277115.2:c.9463G>A:p.Ala3155Thr | rs752191917 | missense_variant | '57/82 | 20:23 | COSV60941441 | Het | Het | Het | -- | Het | Het |
| CFH | chr1:196697553:G:A | NM_000186.4:c.2314G>A:p.Asp772Asn | rs374704701 | missense_variant | '15/22 | 1:22 | COSV66408069 | Het | Het | Het | -- | Het | Het |

**Suppl Table 4. COSMIC not-reported germline mutation loci (SNVs) in the 5 pedigree members (MTF_22, MTF_23, MTF_35, MTF_43, and MTF_44)**

| **Gene_Symbol** | **Chr_Start_Ref_Alt** | **HGVS** | **dbSNPID** | **Mutation_Type** | **Exon** | **VarCards** | **COSMIC** | **NCG_cancerdrivers_Gene** | **MTF_22** | **MTF_23** | **MTF_35** | **MTF_36** | **MTF_43** | **MTF_44** |
| --- | --- | --- | --- | --- | --- | --- | --- | --- | --- | --- | --- | --- | --- | --- |
| CCR8 | chr3:39374553:T:C | NM_005201.4:c.731T>C:p.Val244Ala | . | missense_variant | '2/2 | 19:23 | -- | -- | Het | Het | Het | -- | Het | Het |
| ARFGEF2 | chr20:47601991:A:G | NM_006420.3:c.2117A>G:p.Glu706Gly | rs753947672 | missense_variant | '16/39 | 17:23 | -- | -- | Het | Het | Het | -- | Het | Het |
| DNAH1 | chr7:21818702:G:A | NM_001277115.2:c.9463G>A:p.Ala3155Thr |  | missense_variant | '70/78 | 13:23 |  |  | Het | Het | Het | -- | Het | Het |
| HERC2 | chr3:52429599:G:A | NM_015512.5:c.11164G>A:p.Glu3722Lys |  | missense_variant | 46/93 | 11:23 | -- | HERC2 | Het | Het | Het | -- | Het | Het |
| PLEKHG7 | chr12:93147865:G:A | NM_001377329.1:c.1252-1G>A:. | rs749882326 | splice_acceptor_variant | ' | 10:11 | -- | -- | Het | Het | Het | -- | Het | Het |
| KLF18 | chr1:44604720:T:TCTGGTTACCAGTGGAGGTCATCATATTCTGCCCCCCGTAGAGGGC | NM_001358438.1:c.2583_2584insGCCCTCTACGGGGGGCAGAATATGATGACCTCCACTGGTAACCAG:p.Gln861_Asn862insAlaLeuTyrGlyGlyGlnAsnMetMetThrSerThrGlyAsnGln | rs368966417 | inframe_insertion | '1/2 | . | -- | -- | Hom | Hom | Hom | Hom | Hom | Hom |
| CCT3 | chr1:156290816:A:T | NM_001008800.3:c.309T>A:p.Ser103Arg | rs745486223 | splice_region_variant | '5/12 | 13:22 | -- | -- | Het | Het | Het | -- | Het | Het |
| EFHC1 | chr6:52358163:C:CATAT | NM_001172420.2:c.*1041_*1044dup:. | rs59794069 | 3_prime_UTR_variant | '12/12 | . | -- | -- | Hom | Het | Het | Het | Het | Het |
| NBPF15 | chr1:148558288:T:G | NM_001170755.3:c.-1064+2T>G:. | rs201457826 | splice_donor_variant | ' | . | -- | -- | Het | Het | Het | Het | Het | Het |
| NBPF15 | chr1:148558287:G:C | NM_001170755.3:c.-1064+1G>C:. | rs200419011 | splice_donor_variant | ' | . | -- | -- | Het | Het | Het | Het | Het | Het |
| ECHDC3 | chr10:11805319:CAGG:C | NM_024693.5:c.694_696del:p.Glu232del | rs1225154582 | inframe_deletion | '5/5 | 0:00 | -- | -- | Het | Het | Het | -- | Het | Het |
| ZNF831 | chr20:57767172:TTCGGAGCACAGCGCCGAG:T | NM_001384354.1:c.1101_1118del:p.His369_Glu374del | rs748601113 | inframe_deletion | '4/8 | 0:00 | -- | ZNF831 | Het | Het | Het | -- | Het | Het |
| CTSE | chr1:206329053:C:A | NM_001317331.2:c.652C>A:p.Pro218Thr | rs28548020 | missense_variant | '6/7 | 2:08 | -- | -- | Het | Het | Het | -- | Het | Het |
| ARHGEF11 | chr1:156933085:T:C | NM_001377418.1:c.1038A>G:p.Ile346Met | rs1169114393 | missense_variant | '13/41 | 8:23 | -- | ARHGEF11 | Het | Het | Het | -- | Het | Het |
| IGFBP5 | chr2:217543614:G:C | NM_000599.4:c.526C>G:p.Arg176Gly | rs763073487 | missense_variant | '2/4 | 8:23 | -- | -- | Het | Het | Het | -- | Het | Het |
| ZNF362 | chr1:33742072:G:T | NM_001370212.1:c.226G>T:p.Ala76Ser | rs746967426 | missense_variant | '3/8 | 6:23 | -- | ZNF362 | Het | Het | Het | -- | Het | Het |
| MTSS1L | chr16:70708397:C:T | NM_138383.3:c.865G>A:p.Gly289Arg | rs781080424 | missense_variant | '11/15 | 6:23 | -- | -- | Het | Het | Het | -- | Het | Het |
| HNRNPH1 | chr5:179050060:A:T | NM_001257293.2:c.75T>A:p.Asp25Glu | rs115000818 | missense_variant | '2/14 | 3:23 | -- | -- | Het | Het | Het | -- | Het | Het |

**Suppl Table 5. COSMIC-reported common somatic mutation loci (SNVs) in 2 cancer patients: MTF_22T and MTF_23T**

| **Chr** | **Start** | **End** | **Ref** | **Alt** | **Gene_Symbol** | **avsnp150** | **cosmic96_coding** | **NCG_cancerdrivers_Gene** | **Cosmic CancerGeneCensus Gene** | **NCG cancerdrivers Gene** | **OncoKB Cancer Annotated** | **OncoKB Cancer Suppressor Gene** | **OncoKB Cancer Drug Gene** | **OncoKB Cancer Drug Level** | **OncoKB Cancer_Drug CancerType** | **MTF_22T Tumor_Depth_Fre** | **MTF_23T Tumor_Depth_Fre** | **MTF_43T Tumor_Depth_Fre** |
| --- | --- | --- | --- | --- | --- | --- | --- | --- | --- | --- | --- | --- | --- | --- | --- | --- | --- | --- |
| chr6 | 117690561 | 117690561 | A | C | ROS1 | . | COSV63861329 | ROS1 | ROS1 | ROS1 | Yes | No | ROS1 | 1,1,2,2,3 | Non-Small Cell Lung Cancer | 0.115 | 0.035 | . |
| chr6 | 152280130 | 152280130 | C | T | ESR1 | rs200263274 | COSV52782246 | ESR1 | ESR1 | ESR1 | Yes | No | ESR1 | 1,2,3 | Breast Cancer | 0.25 | 0.086 | . |
| chr7 | 116385784 | 116385784 | G | T | MET | rs374470641 | COSV59268514 | MET | MET | MET | Yes | No | MET | 1,1,2,2,2,2,3,3,4,R2,R2,R2 | Non-Small Cell Lung Cancer | 0.117 | 0.057 | . |
| chr11 | 33887921 | 33887921 | T | C | LMO2 | . | COSV57647457 | LMO2 | LMO2 | LMO2 | Yes | No | LMO2 | Dx2 | T-Lymphoblastic Leukemia/Lymphoma | 0.177 | 0.059 | . |
| chr14 | 68897189 | 68897189 | G | T | RAD51B | . | COSV66852041 | RAD51B | RAD51B | RAD51B | Yes | Yes | RAD51B | 1 | Prostate Cancer | 0.177 | 0.102 | . |
| chr11 | 118339759 | 118339759 | A | G | KMT2A | . | COSV63293378 | KMT2A | KMT2A | KMT2A | Yes | Yes | KMT2A | 3,3,Dx1,Dx1,Dx1,Dx2,Dx2,Dx2,Px1,Px1 | Acute Myeloid Leukemia,B-Lymphoblastic Leukemia/Lymphoma | 0.198 | 0.106 | . |
| chr2 | 213100450 | 213100450 | T | A | ERBB4 | rs979181492 | COSV53596369 | ERBB4 | ERBB4 | ERBB4 | Yes | No | . | . | . | 0.188 | 0.099 | . |
| chr2 | 213392465 | 213392465 | C | A | ERBB4 | . | COSV53583307 | ERBB4 | ERBB4 | ERBB4 | Yes | No | . | . | . | 0.106 | 0.047 | . |
| chr7 | 124542438 | 124542438 | - | G | POT1 | . | COSV62932859 | POT1 | POT1 | POT1 | Yes | Yes | . | . | . | 0.096 | 0.08 | . |
| chr9 | 123867258 | 123867258 | C | G | CNTRL | rs958683954 | COSV53053157 | CNTRL | CNTRL | CNTRL | Yes | No | . | . | . | 0.188 | 0.061 | . |
| chr22 | 19264459 | 19264459 | C | T | CLTCL1 | rs192480548 | COSV54239655 | CLTCL1 | CLTCL1 | CLTCL1 | Yes | No | . | . | . | 0.167 | 0.047 | . |

**Suppl Table 6. Common somatic mutation loci (SNVs) in 2 cancer patients: MTF_23T and MTF_43T**

| **Chr** | **Start** | **End** | **Ref** | **Alt** | **Gene_Symbol** | **avsnp150** | **cosmic96_coding** | **NCG_cancerdrivers_Gene** | **Cosmic CancerGeneCensus Gene** | **NCG cancerdrivers Gene** | **OncoKB Cancer Annotated** | **OncoKB Cancer SuppressorGene** | **OncoKB Cancer Drug Gene** | **OncoKB Cancer Drug Level** | **OncoKB Cancer_Drug CancerType** | **MTF_22T_Tumor_Depth_Fre** | **MTF_23T_Tumor_Depth_Fre** | **MTF_43T_Normal_Depth_Fre** |
| --- | --- | --- | --- | --- | --- | --- | --- | --- | --- | --- | --- | --- | --- | --- | --- | --- | --- | --- |
| chr8 | 120772929 | 120772930 | CA | - | TAF2 | . | . | . | . | . | . | . | . | . | . | . | 0.034 | 0.02 |
| chr2 | 74601748 | 74601752 | GCGGT | - | DCTN1 | . | . | DCTN1 | DCTN1 | DCTN1 | No | No | . | . | . | . | 0.037 | 0.026 |
| chr10 | 63662456 | 63662456 | G | A | ARID5B | rs566045630 | COSV54414236 | ARID5B | . | ARID5B | Yes | Yes | . | . | . | . | 0.058 | 0.03 |
| chr3 | 33714951 | 33714951 | G | A | CLASP2 | . | COSV56287197 | CLASP2 | . | CLASP2 | . | . | . | . | . | . | 0.433 | 0.054 |
| chr10 | 68294239 | 68294239 | T | C | CTNNA3 | rs963698560 | . | CTNNA3 | . | CTNNA3 | . | . | . | . | . | . | 0.13 | 0.027 |
| chr12 | 44491628 | 44491628 | A | C | TMEM117 | rs150272754 | COSV56908968 | . | . | . | . | . | . | . | . | . | 0.097 | 0.024 |
| chr6 | 35971595 | 35971595 | C | G | SLC26A8 | rs199745529 | . | . | . | . | . | . | . | . | . | . | 0.066 | 0.028 |
| chr12 | 21121995 | 21122003 | GTCTGGGAG | - | SLCO1B3-SLCO1B7 | . | . | , | , | , | , | , | , | , | , | . | 0.036 | 0.024 |
| chr17 | 26384036 | 26384036 | A | C | NLK | . | . | . | . | . | . | . | . | . | . | . | 0.041 | 0.024 |
| chr19 | 40788472 | 40788488 | ATAGGACAGTCACCTCT | - | MIR641 | . | . | . | . | . | . | . | . | . | . | . | 0.053 | 0.021 |
| chr22 | 50757352 | 50757353 | CA | - | DENND6B | . | . | . | . | . | . | . | . | . | . | . | 0.038 | 0.039 |

**Suppl Table 7. Common somatic CNVs in 3 patients: MTF_22T, MTF_23T and MTF_43T**

| **Chromosome** | **MTF_22T_overlap_range** | **MTF_22T_overlap_len** | **MTF_23T_overlap_range** | **MTF_23T_overlap_len** | **MTF_43T_overlap_range** | **MTF_43T_overlap_len** | **Type** | **All protein coding genes** |
| --- | --- | --- | --- | --- | --- | --- | --- | --- |
| chr14 | 20447232_20447231 | 300000 | 20447232_20447231 | 300000 | 20200001_20400000 | 200000 | DUP | OR4Q3, OR4M1, OR4N2, OR4K3, OR4K2, OR4K5, OR4K1, OR4K15, OR4Q2, OR4K14 |
| chr6 | 57200001_57400000 | 200000 | 57200001_57400000 | 200000 | 57200001_57400000 | 200000 | DUP | PRIM2 |

**Suppl Table 8. Differences in DNA methylation between the twins MTF_43 and MTF_44**

| **Gene Name** | **element** | **Direction** | **Cosmic Genes** | **Cosmic-Cancer Gene Census** | **Cosmic-Role in Cancer** |
| --- | --- | --- | --- | --- | --- |
| WT1 | CGI | Hyper | Yes | Yes | TSG, oncogene, fusion |
| SIX1 | CGI | Hypo | Yes | Yes | oncogene |
| GNAS | Promoter | Hypo | Yes | Yes | oncogene |
| PAX7 | CGI | Hypo | Yes | Yes | fusion |
| ZNF331 | Promoter | Hypo | Yes | Yes | TSG, fusion |
| SLX4 | CGI | Hypo | Yes | . | . |
| ADGRA2 | CGI | Hypo | Yes | . | . |
| RXRA | Promoter | Hypo | Yes | . | . |
| MB21D2 | CGI | Hypo | Yes | Yes |  |

TSG: tumor suppressor gene. MTF_43: leukemia ALL. MTF_44: healthy twin.

# Supplementary Figures

**
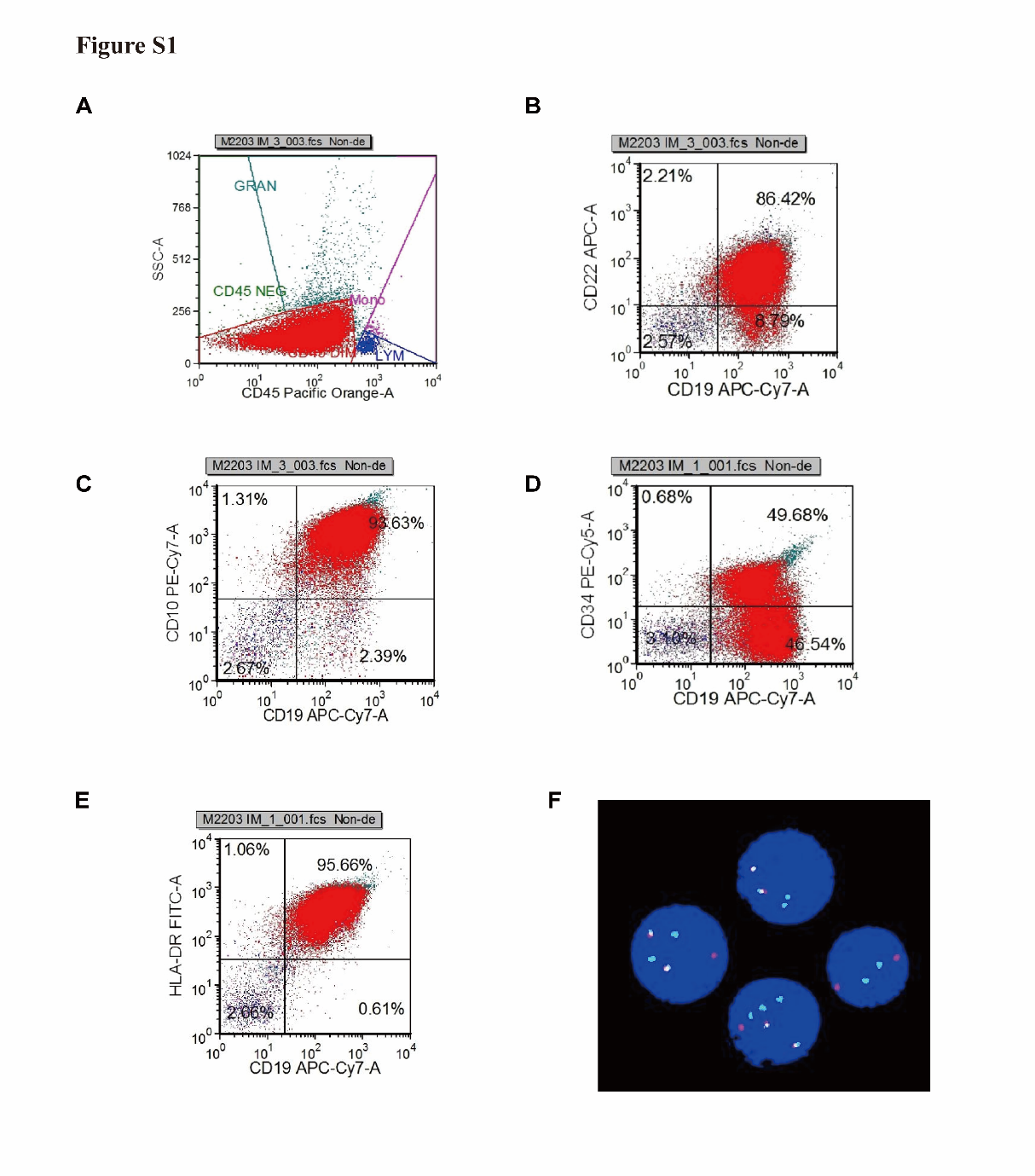
**

**Supplementary Figure 1.** **Flow cytometry and FISH analysis for patients IV-3 diagnosed** **with B-ALL.** (A) B lymphoblasts accounted for approximately 93.6% of the total nucleated cells. (B, C, D, E) IV-3 B cells highly expressed CD19+ and CD22+ (86.42%), CD19+ and CD10+ (93.63%), CD19+ and CD34+ (49.63%), and CD19+ and HLADR+ (95.66%). (F) Fluorescence in situ hybridization (FISH) using dual fusion probes with ETV6 (red)/RUNX1 (green) on the bone marrow sample at diagnosis from MTF_43T, shows positive for ETV6::RUNX1 gene fusion.


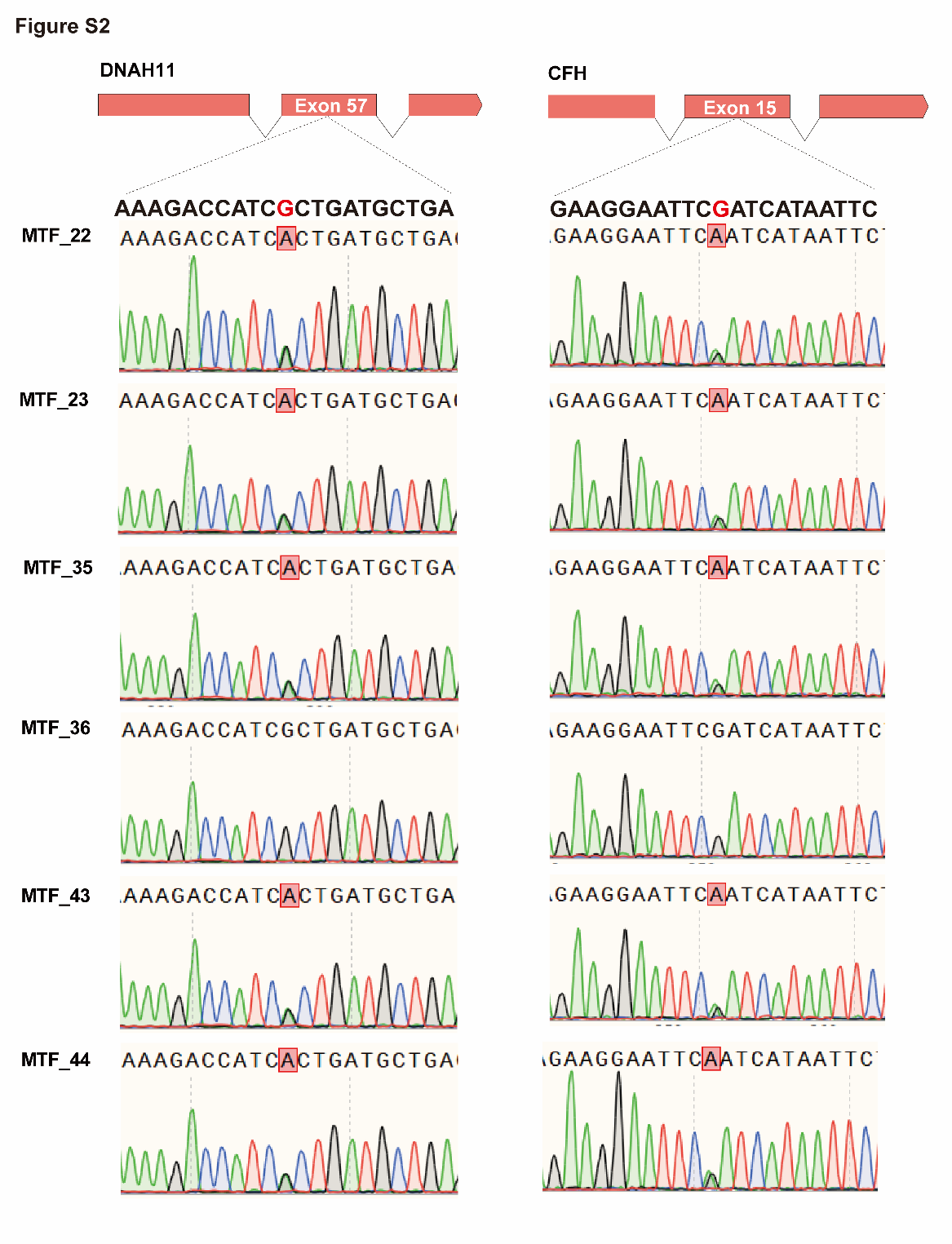


**Supplementary Figure 2.** Sanger sequencing validation of the germline SNVs in the DNAH11 and CFH genes identified in the pedigree. Six family members shared the DNAH11 (NM_001277115.2:c.9463G>A) and CFH (NM_000186.4:c.2314G>A) mutations.


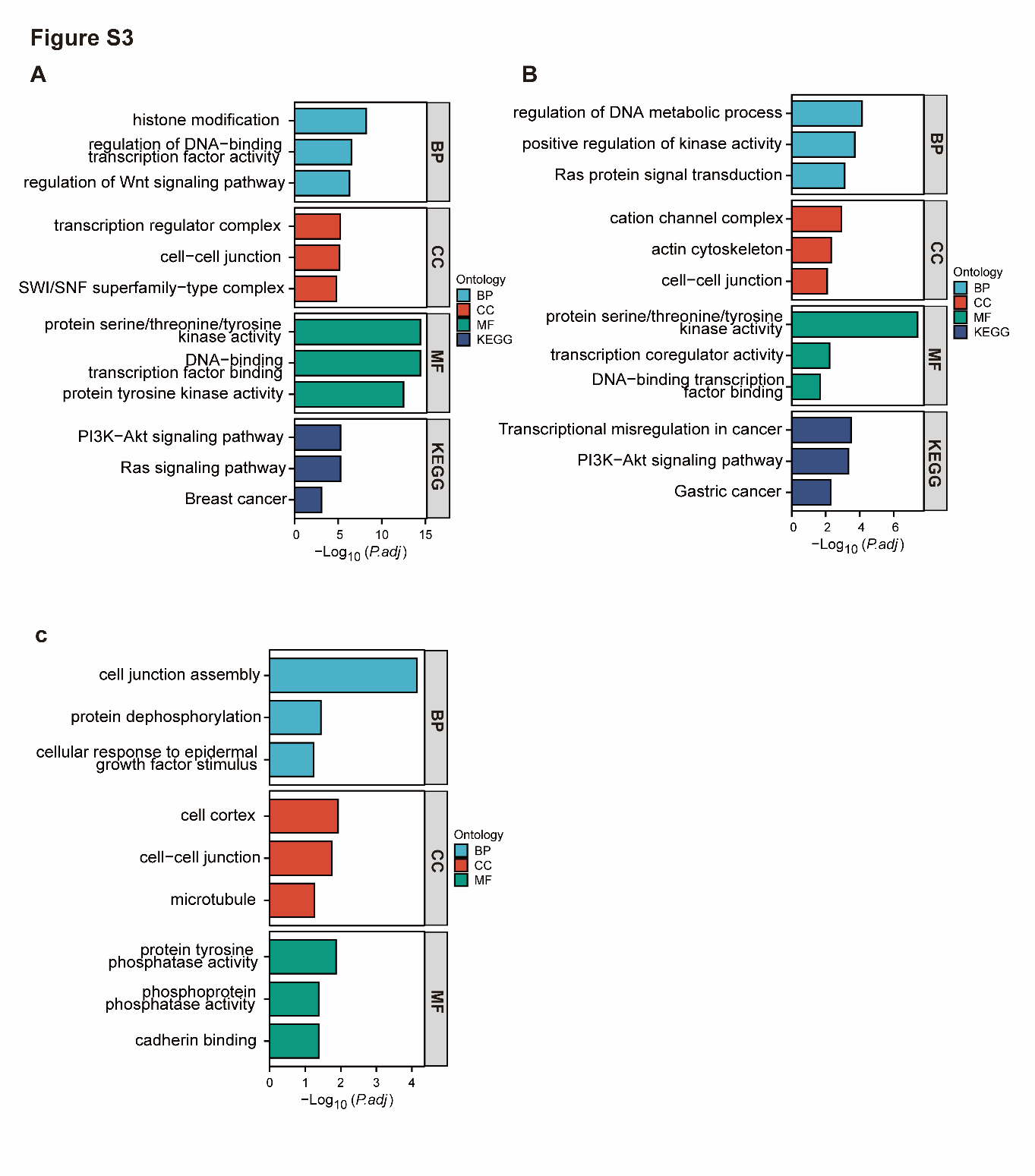


**Supplementary Figure 3.** Functional pathway of the genes with SNVs identified in the three cancer patients. (A) Somatic mutation genes of MTF_22T. (B) Somatic mutation genes of MTF_23T. (C) Somatic mutation genes of MTF_43T.


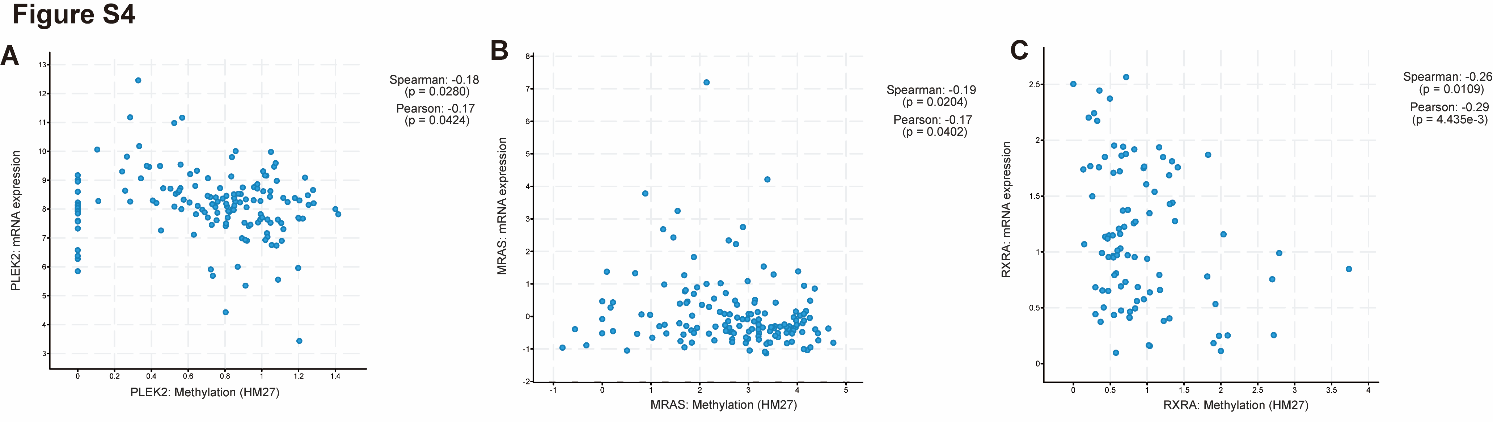


**Supplementary Figure 4.** Correlation analysis of mRNA expression of hypomethylating genes and prognostic analysis with expression levels of three hypomethylating genes. (A)(B)(C) Correlation analysis of mRNA expression level and methylation level of PLEK2, MRAS, and RXRA.
